# Development and Validation of a Novel HPLC Method to Analyse Metabolic Reaction Products Catalysed by the CYP3A2 Isoform: In Vitro Inhibition of CYP3A2 Enzyme Activity by Aspirin (Drugs Often Used Together in COVID-19 Treatment)

**DOI:** 10.3390/molecules27030927

**Published:** 2022-01-29

**Authors:** Amira Hussain, Declan P. Naughton, James Barker

**Affiliations:** School of Life Sciences, Pharmacy and Chemistry, Kingston University, Kingston-upon-Thames, London KT1 2EE, UK; d.naughton@kingston.ac.uk (D.P.N.); j.barker@kingston.ac.uk (J.B.)

**Keywords:** cytochrome P450, dexamethasone, 6β-hydroxydexamethasone, aspirin, competitive inhibitor, CYP3A activity

## Abstract

Aspirin (also known as acetylsalicylic acid) is a drug intended to treat fever, pain, or inflammation. Treatment of moderate to severe cases of COVID-19 using aspirin along with dexamethasone has gained major attention globally in recent times. Thus, the purpose of this study was to use High-Performance Liquid Chromatography (HPLC) to evaluate the in vitro inhibition of CYP3A2 enzyme activity using aspirin in rat liver microsomes (RLMs). In this study, an efficient and sensitive HPLC method was developed using a reversed phase C18 column (X Bridge 4.6 mm × 150 mm, 3.5 µm) at 243 nm using acetonitrile and water (70:30 *v/v*). The linearity (r^2^ > 0.999), precision (<15%), accuracy and recovery (80–120%), limit of detection (5.60 µM and 0.06 µM), limit of quantification (16.98 µM and 0.19 µM), and stability of the newly developed method were validated for dexamethasone and 6β-hydroxydexamethasone, respectively, following International Conference on Harmonization (ICH) guidelines. This method was applied in vitro to measure CYP3A2 activity. The results showed that aspirin competitively inhibits 6β-hydroxylation (CYP3A2 activity) with an inhibition constant (Ki) = 95.46 µM and the concentration of the inhibitor causing 50% inhibition of original enzyme activity (IC_50_) = 190.92 µM. This indicated that there is a minimal risk of toxicity when dexamethasone and aspirin are co-administrated and a very low risk of toxicity and drug interaction with drugs that are a substrate for CYP3A2 in healthcare settings.

## 1. Introduction

Cytochrome P450 enzymes are responsible for the biotransformation of xenobiotics and the metabolism of endogenous compounds [1]. They are mainly found in the liver; however, a number of these enzymes are also expressed in the kidney, small intestine, placenta, and lungs [2]. The synthesis of these enzymes takes place endogenously, and both non-genetic and genetic factors could influence enzyme synthesis.

Many drug–drug interactions of clinical interest change the pharmacokinetic behavior of drugs due to changes in the hepatic metabolic pathway of drugs that are catalysed by CYP450 enzymes [3]. The drug interactions are caused by either CYP450 inhibition or induction. Enzyme induction or inhibition by food, drugs, and medicinal herbs, for instance, can be responsible for changes in the metabolic capacity of these enzymes [4], although inhibition is considered more important in terms of adverse clinical outcomes.

CYP450 enzyme inhibition by drugs could increase the concentration of other metabolizing drugs and could result in drug toxicity issues [5]. CYP3A is the most important family among the identified families of CYP450 enzymes involved in biotransformation and metabolism in humans. The CYP3A family is responsible for the metabolism of 70% of drugs as well as for indicating broad substrate specificity [6]. CYP3A is frequently associated with most drug interactions because this isoform is highly inducible and could potentially be inhibited by other drugs and herbs [6]. Of the various isoforms, CYP3A4 is the most abundant isoform present in human liver microsomes and is responsible for the metabolism of various anticancer drugs [7]. Although CYP3A4 activity exhibits 5–10-fold inter-individual variability, it still has substantial potential for clinical applications [7].

Dexamethasone is a steroidal anti-inflammatory drug that is widely used for the treatment of different conditions such as cancer, autoimmune diseases, and chronic inflammatory diseases. Dexamethasone has been shown to reduce deaths in those who have been hospitalized for severe cases of COVID-19 where patients required ventilator support [8]. The use of dexamethasone to treat multiple diseases significantly increases the risk of drug–drug interactions [9]. Tomlinson et al. (1997) stated that dexamethasone is metabolized into 6 α as a minor metabolite and 6β-hydroxymethasone as a major metabolite through CYP3A4 enzyme activity using human liver microsomes.

Previous studies have shown that ketoconazole, ellipticine, and gestodene cause the inhibition of dexamethasone 6-hydroxylation [10]. Although many studies have been conducted in human liver microsomes, rats have the closest metabolic profile to humans and show 71% sequence homology [11]. The CYP3A2 isoform in male-specific rat liver microsomes (RLMs) is responsible for the 6-hydroxylation of dexamethasone (corticosteroid) [11]. Wang et al. [12] stated that codeine administration in male rats can inhibit the metabolism of midazolam (CYP3A2 activity).

**Figure 1:**
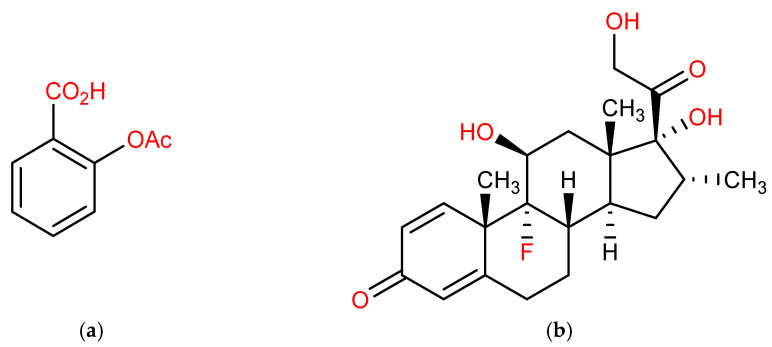
(**a**) Aspirin (inhibitor); (**b**) dexamethasone (substrate).

COVID-19 infection has rapidly grown into a worldwide pandemic, and this has had a significant impact on human health. Dexamethasone and aspirin (Figure 1) are some of the drugs being used for the treatment of COVID-19 during the pandemic. The combined use of aspirin and dexamethasone has been shown to reduce the symptoms in moderate to severe COVID-19 infection [13]. A recent study has provided evidence in support of primary healthcare centres where they used aspirin and dexamethasone for the therapeutic management of severe COVID-19 patients [14]. Nonsteroidal anti-inflammatory drugs (NSAIDs) and aspirin are the most used therapeutic drugs worldwide [15]. Aspirin is an O-acetyl derivative of salicylic acid (acetylsalicylic acid). It is believed that it transfers this acetyl group to the amino (-NH_2_) and hydroxy (-OH) functionalities present in biological molecules [16]. Aspirin is also a prostaglandin synthase inhibitor that inhibits the production of prostaglandins. It has a non-selective effect on the cyclooxygenase-1 (COX-1) and cyclooxygenase-2 (COX-2) enzymes [17].

In this systematic study, a HPLC method was developed and validated to investigate the impact of aspirin on the activity of the CYP3A2 enzyme in rat liver microsomes, whereby dexamethasone was the substrate. This study promotes the safe administration of COVID-19 drugs (dexamethasone and aspirin) in clinical practice.

## 2. Results and Discussion

### 2.1. HPLC Method Development

The analytical method for the enzymatic assay was optimised and evaluated using the following chromatographic conditions: mobile phase consisting of acetonitrile and water (70% acetonitrile and 30% H_2_O, *v/v*) with an injection volume of 10 µL, a flow rate of 0.6 mL/min, column temperature set at 25 °C, and a run time of 8 min. The chosen wavelength (λ) was 243 nm [10]. The selected internal standard was 4-hydroxyoctanophenone.

### 2.2. HPLC Method Validation

#### 2.2.1. Linearity and Range

According to the ICH guidelines [18], the linearity was tested using different concentrations (0, 25, 50, 100, 150, and 200 µM) of working solutions of dexamethasone and 6β-hydroxydexamethasone (0, 0.2, 0.4, 0.6, 0.8, and 1 µM). Working solutions were prepared in the mobile phase (70% acetonitrile and 30% H_2_O, *v/v*) and injected into the HPLC for the construction of the calibration curve. The mean peak area obtained from the HPLC chromatograms was plotted against the concentrations of each analyte (dexamethasone and 6β-hydroxydexamethason) to assess the calibration graph (Figure S1). All of the obtained data were corrected for the internal standard. Table 1 represents the results of the linearity study. The r^2^ values were within the acceptable ICH criterion (r^2^ > 0.99), and the relative standard deviation at each concentration (%RSD < 10%) met the criteria of the ICH guidelines.

#### 2.2.2. Specificity and Selectivity

**Figure 2:**
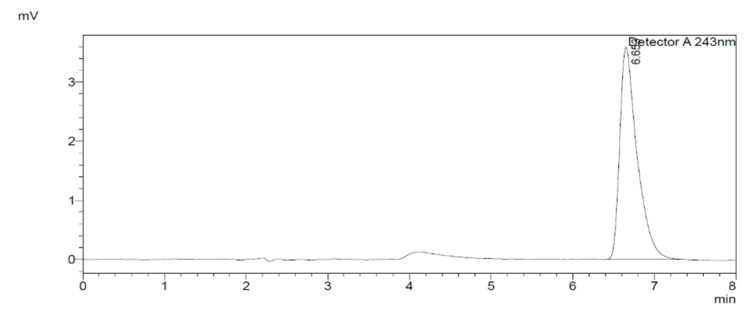
Negative control blank shows the specificity of the method.

**Figure 3:**
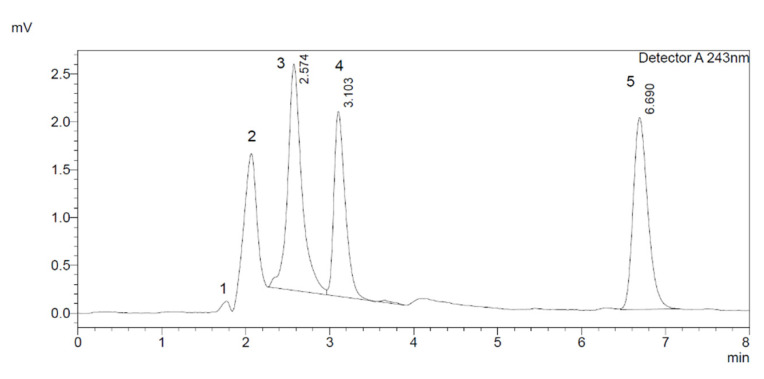
HPLC profile of CYP3A2 assay components. (1) NADPH (Nicotinamide Adenine Dinucleotide Phosphate Hydrogen)-regenerating system, (2) aspirin, (3) 6β-hydroxydexamethasone, (4) dexamethasone, and (5) 4-hydroxyoctanophenone (IS).

Specificity/selectivity was evaluated by running the diluent blank (70% acetonitrile and 30% H_2_O, *v/v*) and internal standard solution (15 µM) in a 1 mL HPLC vial to check that the outcomes of the analytical method were not altered by the drugs’ constituents (Figure 2). Figure 3 shows the separation of the enzyme peak (NADPH (Nicotinamide Adenine Dinucleotide Phosphate Hydrogen-regenerating system) from the inhibitor (aspirin 200 µM), metabolite (6β-hydroxydexamethasone), dexamethasone, and internal standard.

#### 2.2.3. Limit of Detection (LOD) and Limit of Quantification (LOQ)

The sensitivity of the analytical method was achieved by an evaluation of its LOD and LOQ. The lowest detectable concentration of the analyte is LOD. The minimum measurable concentration in the analytical method and that can be determined quantitatively with suitable precision is LOQ. The standard deviation (σ) method was used to determine LOD and LOQ using the following formulae:

LOD = (3.3 × σ/S)(1)

LOQ = (10 × σ/S)(2)

where S is the slope of the calibration curve.

The values of LOD and LOQ for dexamethasone and 6β-hydroxydexamethasone are presented in Table 2.

#### 2.2.4. Precision

##### Intraday Precision of Dexamethasone

The intraday method precision was determined by analysing standard samples in triplicate at three different concentrations levels, i.e., low (40 µM), medium, (110 µM), and high (185 µM), of dexamethasone. The results are summarised in Table 3. The percentage relative standard deviation (%RSD) was <5% for dexamethasone, and recovery values were found to be within the range of the ICH acceptance criterion (80–120%). The outcomes revealed that there is no large variation in the concentration of dexamethasone in the intraday analysis. The obtained values were considered satisfactory for the planned use of the method.

##### Interday Precision of Dexamethasone

The interday precision was determined by injecting standard sample solution for three consecutive days at three concentration levels (low (40 µM), medium, (110 µM), and high (185 µM)). Each sample was run in triplicate. The results are summarised in Table 4, which shows that the recovery values are within the range of the ICH guidelines (80–120%), and the percentage relative standard deviation (%RSD) was <5% for dexamethasone. The outcomes of the experiment revealed that there is no large variation in the interday analysis.

##### Intraday Precision of 6β-Hydroxydexamethasone

The intraday precision of 6β-hydroxydexamethasone was assessed by measuring the low (0.3 µM), medium, (0.5 µM), and high (0.85 µM) concentrations in triplicate in a batch experiment. The results are shown in Table 5 and illustrate that the recovery values (80–120%) are within the range of the ICH guidelines, and the percentage-relative standard deviation values (% RSD) are <0% for 6βh-hydroxydexamethasone. The experiment outcomes revealed that there is no large variation present between the concentration of the 6β-hydroxydexamethasone samples in the intraday precision analysis.

##### Interday Precision of 6β-Hydroxydexamethasone

Interday precision was evaluated by measuring the 6β-hydroxydexamethasone standard solution at three concentrations levels (low (0.3 µM), medium, (0.5 µM), and high (0.85 µM) for three consecutive days. The results of the experiment are shown in Table 6. The %RSD (relative standard deviation) is <10% for 6β-hydroxydexamethasone, and recovery values are within the range of the ICH guidelines (80–120%). The experimental data revealed that no large variations are present in terms of inter-assay precision.

#### 2.2.5. Stability Study

##### Solution Stability of Substrate (Dexamethasone)

For stability testing, dexamethasone calibration curves were run for intraday and interday measurements at three concentration levels (40, 110, and 185 µM) at ambient temperature. Stability samples were analyzed in triplicate. The results of the stability tests are shown in Table 7.

The outcomes of the interday and intraday stability checks indicate that there was no variation in the concentration of dexamethasone. In the intraday stability analysis, the concentration of dexamethasone after 10 h was the same compared to the initial concentrationat at 0 h, whereas in the interday analysis, the chromatographic behaviour of dexamethasone remained the same at days 1, 2, and 3 compared to the initial concentrations. The r^2^ value obtained from the calibration curves of the intermediate (intraday) analysis was 0.9984 (in accordance with ICH guidelines). The calibration curve for the repeatability analysis was averaged, and the curve equation for dexamethasone was y = 0.277x + 0.6419, where the least regression square values (r^2^) were within the ICH guidelines (0.9981). Linear equations were further used to calculate the dexamethasone concentrations (low, medium, and high) and their % recovery. The results reveal that the % recovery and accuracy values remained within the range of the ICH guidelines (80–120%). Furthermore, no substantial degradation within the day (intraday) and between days (interday) was observed (Table 7), thus showing that dexamethasone was stable for up to three days at ambient temperature, which is in accordance with the stability study performed by Heda et al. (2011) [19].

##### Solution Stability of 6β-Hydroxydexamethasone (Metabolite)

An evaluation of the stability of the working standard solutions of 6β-hydroxydexamethasone was performed at ambient temperature by intraday and interday analysis at three concentration levels (low (0.3 µM), medium (0.5 µM), and high (0.85 µM)). Experiments were performed in triplicate, and the results are summarized below in Table 8.

The results of the 6β-hydroxydexamethasone stability test showed that there were no obvious changes perceived in the chromatographic behavior and elution profile of the metabolite. In the intraday stability analysis, the chromatographic behaviour of 6β-hydroxydexamethasone after 10 h remained the same compared to the initial concentration at 0 h whereas, in the interday analysis, the concentration of 6β-hydroxy dexamethasone stayed the same at days 1, 2, and 3, compared to the initial concentrations. The calibration curve was plotted for intermediate analysis, and the straight-line equation was y = 2.4559x + 0.0767. The averaged calibration curve of the metabolites was constructed using three days of calibration data, and the averaged straight-line equation was (y = 2.0023x + 0.0364). All of the the % recovery and % accuracy values were in the range specified by the ICH guidelines (80–120%), demonstrating the fact that the metabolite solution was stable. Therefore, the results indicate that 6β-hydroxydexamethasone solution was stable at ambient temperature during intraday and interday analysis, which is in accordance with the literature [7]. It is evident that metabolite sample solutions need to be kept and run on HPLC for longer than overnight for the incubation process to see the inhibition.

#### 2.2.6. Robustness of the Method

The robustness of the method (50 µM aspirin, 0.2 µM 6β-hydroxydexamethasone, 25 µM dexamethasone and 15 µM internal standard) was tested by changing the following parameters: increasing the wavelength by 5 nm, increasing the temperature by 5 °C, and increasing the flowrate. Therefore, replicate injections (*n* = 3) of standard multianalyte solution were performed. The observations were made based on peak areas and changes in the retention time. Table 9 summarises the effects of the wavelength, temperature, and flow rate variation on the peak area and retention time of the compounds.

The outcomes of the robustness test showed that the developed method could optimally perform when small changes are made to parameters such as the wavelength, temperature, and flow rate. Good dexamethasone, 6β-hydroxydexamethasone, and internal standard separation were achieved for all of the temperature, wavelength, and flow rate variations.

### 2.3. Optimisation of Incubation Time for Incubation System In Vitro

**Figure 4:**
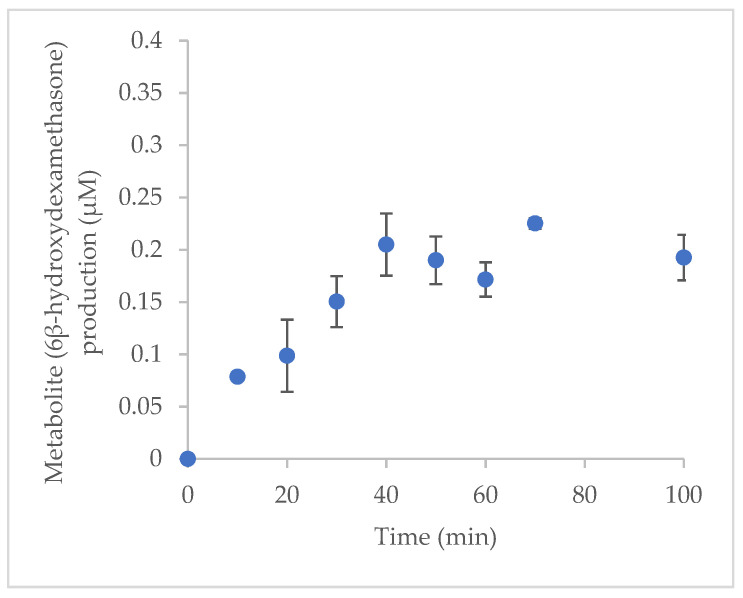
Effects of incubation time on 6β-hydroxydexamethasone production.

The incubation time was optimised (Figure 4). The 6β-hydroxydexamethasone formation rate from dexamethasone (50 µM) by cytochrome P3A2 was linear and took place over 40 min. Thus, the optimal incubation time for CYP3A2 was 40 min.

### 2.4. Inhibitory Effects of Aspirin on CYP3A2 Activity in Rat Liver Microsomes

**Figure 5:**
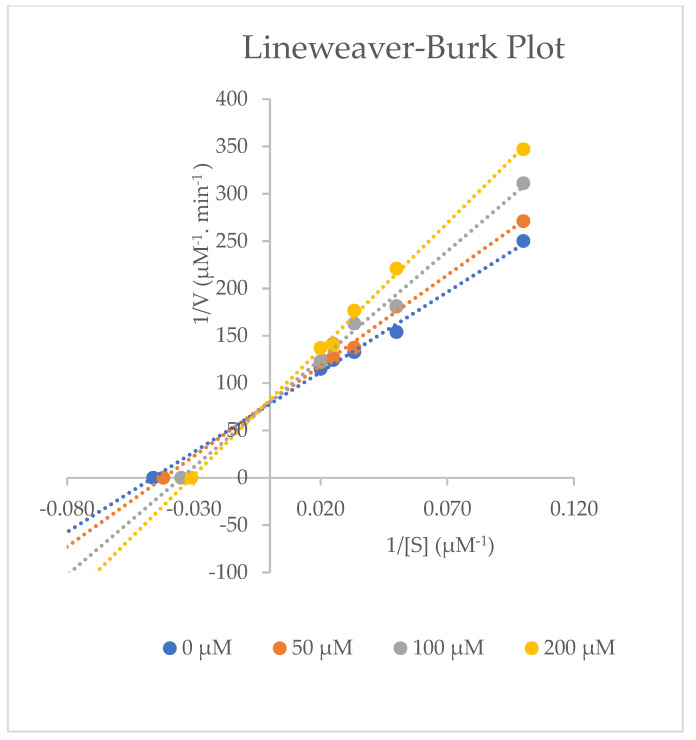
Representative Lineweaver–Burk plot for CYP3A2 enzyme inhibition on dexamethasone metabolism into 6β-hydroxydexamethasone with 0, 50, 100, and 200 µM aspirin in rat liver microsomes. Average data are taken from triplicate measurements.

**Figure 6:**
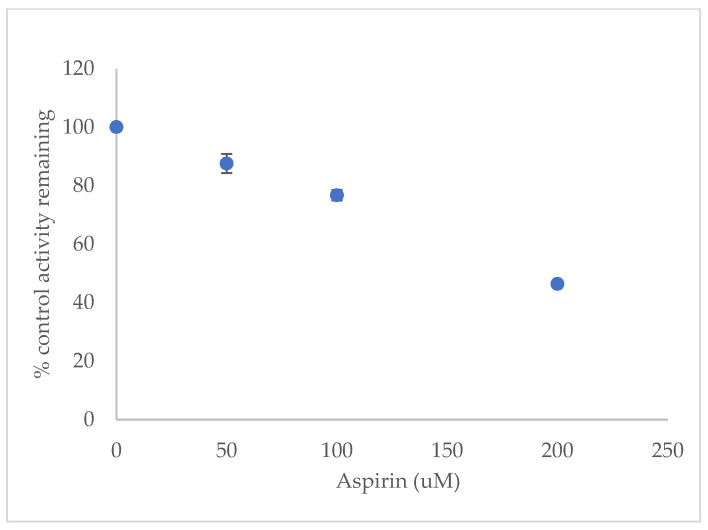
Effect of aspirin on CYP3A2 activity in rat liver microsomes. Control activity was taken as 100%. Average data are taken from triplicate measurements.

To see the effects of aspirin on rat CYP activities, different concentrations of dexamethasone (10, 20, 30, 40, and 50 µM) in the presence of 0, 50, 100, and 200 µM aspirin were investigated. Microsomal proteins (0.5 mg/mL) were incubated for 40 min at 37 °C with dexamethasone, 3.0 mM magnesium chloride (MgCl_2_), 1.0 mM Ethylenediaminetetraacetic acid (EDTA), 1.0 mM Nicotinamide Adenine Dinucleotide Phosphate Hydrogen (NADPH), and 0.067 M phosphate buffer (pH 7.4). Aspirin competitively inhibited the production of 6β-hydroxydexamethasone, as presented in Figure 5 and Table 10, whereas the effect of aspirin on 6β-hydroxydexamethasone production when using 10–50 µM substrate is shown in Figure 6. Aspirin (0–200 µM), even at lower than therapeutically relevant concentrations (150–300 µg/mL), causes a 50% inhibition of CYP3A2 enzyme activity (IC_50_), as presented in Table 10.

The inhibition of hepatic cytochrome P450 activity is one of the most significant mechanisms of a drug interaction. Severe adverse events have been associated with drug interactions caused during coadministration [20]. Thus, regulatory authorities need to conduct interaction studies both in vitro and in vivo during drug development.

Aspirin and dexamethasone have been used for the treatment of COVID-19 during the pandemic (2020), and the associated use of aspirin and dexamethasone causes a reduction in COVID-19 mortality. This research is the first to investigate the effects of aspirin on the metabolism of dexamethasone (CYP3A2 enzyme activity).

This study has demonstrated that dexamethasone is metabolised in rat liver microsomes to 6β-hydroxydexamethasone. The in vitro findings indicated that aspirin at doses between 0–200 µM acts as a competitive inhibitor and may weakly inhibit cytochrome P4503A2 enzyme activity. The V_max_ (V_max_ is the maximum rate of a reaction catalysed by an enzyme) for the inhibition experiments at three aspirin concentration levels (50, 100, and 200 µM) remained the same as it did for negative control (without Inhibitor). Whereas higher K_m_ (the concentration of substrate requiring the half-maximal activity of the enzyme) values were obtained in the presence of the inhibitor, as shown in Table 10. Alpha prime (α’) indicates the effect of an inhibitor on an enzyme’s affinity for its substrate, and similarly, the effect of the substrate on the enzyme’s affinity for the inhibitor. As indicated in Table 10, the α’ values of aspirin are greater than 1.0 (competitive inhibitor) [21]. The CL_int_ (the intrinsic ability of hepatic CYP450 enzymes to metabolise the drug) of drugs is often predicted based on in vitro data obtained from kinetic analysis (Michaelis–Menten). It was calculated as a ratio of in vitro kinetic constants V_max_ and K_m_ [22], as presented in Table 10.

The results show that aspirin is a competitive inhibitor that binds to the active site of the CYP3A2 enzyme and decreases the activity of the CYP3A2 isoform, which is responsible for the 6β-hydroxylation of dexamethasone in male rats, with the K_i_ (the binding affinity between the enzyme and inhibitor) = 95.46 ± 4.25 µM and IC_50_ (inhibitor concentration required to inhibit 50% of the enzyme activity) = 190.92 ± 8.50 µM. A compound with an IC_50_ value below 1 µM is considered to be a strong inhibitor, and it is considered to be a weak inhibitor if the IC_50_ value is more than 50 µM [23]. Therefore, aspirin has a weak inhibitory effect on CYP3A2 isoform activity.

These in vitro findings would be useful for future in vivo studies in the healthcare sector. The simple metabolic profile of dexamethasone shows that this steroid could be useful as an in vivo probe for CYP3A4 [10]. Further in vitro and in vivo clinical studies on the potential risks associated with the interactions of dexamethasone and aspirin in humans are required.

## 3. Materials and Methods

### 3.1. Chemicals

The compound 6β-Hydroxydexamethasone was purchased from Cayman Chemical Company (Ann Arbor, MI, USA). Dexamethasone from Tokyo Chemical Industry CO.LTD (Tokyo, Japan), and 4′-hydroxyoctanophenone with a purity greater than 99% were obtained from Alfa Aesar (A Johnson Matthey Company, London, UK). Potassium phosphate monobasic, potassium phosphate dibasic, glucose-6-phosphate (G-6-P), glucose-6-phosphate dehydrogenase (G-6-PDH), phosphoric acid (85% *w/w*), NADP^+^ (Nicotinamide Adenine Dinucleotide Phosphate), EDTA (Ethylenediaminetetraacetic acid), and magnesium chloride (MgCl_2_) were purchased from Merck. Diethyl ether was purchased from Fischer Scientific (Bishop Meadow Road, Loughborough, UK), and ethyl acetate was purchased from VWR chemicals (France). HPLC-grade acetonitrile and water were obtained from Merck, Co. (Old Brickyard, Gillingham, UK).

The pooled liver microsomes from male rats (Sprague Dawley) were stored at −80 °C and were purchased from Merck (Old Brickyard Road, Gillingham, UK).

### 3.2. Instruments

A 570 pH Meter from JANEWAY Limited (Beacon Road, Stone, Staffordshire, ST15 0SA, UK) was purchased. A high-performance liquid chromatographic system (LC-2010A HT Shimadzu Corporation, Kyoto, Japan) was used for the analysis and was equipped with a degasser, a UV detector, a low-pressure quaternary pump, a LC column oven, and an autosampler. A WATERS (Waters Corporation, 34 Maple St., Milford, MA, USA) C18 column (15 mm × 4.6 mm, 3.5 µm particle size) was used for chromatographic separation. The chromatographic data were processed using Shimadzu HPLC 1 LabSolutions (software processing system). The shaking incubator used for the incubation of the tubes was from Eppendorf Ltd. (Stevenage, UK).

### 3.3. Cytochrome P450 Assay

#### 3.3.1. Dexamethasone 6β-Hydroxylation Assay for CYP3A2

Assay development and validation were carried out using a LC-2010A HT Module HPLC system (Shimadzu, Toyko, Japan). Target components (4-hydroxyoctanophenone used as an internal standard, aspirin as the inhibitor, dexamethasone as the CYP3A2 substrate, and 6β-hydroxydexamethasone as the CYP3A2 metabolite) were separated on a C18 column.Good separation of the compounds of interest was achieved using the optimised acetonitrile/water (70%/30%, *v/v*) mobile phase. The retention time (t_R_) of the four compounds is shown in Table 11. The chromatographic separation was performed at 25 °C and at a flow rate of 0.6 mL/min. A 10 µL solution volume was injected for HPLC analysis, and all the components were detected at the 243 nm wavelength. Table 11 provides information concerning efficiency (N), where N = 5.54 × (t_R_/W_½_) and W_½_ = width at half peak height; plate height (H), where H(cm) = column length/N; asymmetry factor (AsF), where AsF = B/A, and A is the distance from the leading edge of the peak to the midpoint of the peak measured at 10% of peak height, and B is the distance from the midpoint of the peak to the trailing edge of the peak measured at 10% of the peak height; and resolution (R_s_), where Rs = 2∆t_R_/0.5(W_1_ + W_2_), and W is the width at the peak base. As can be seen from Table 11, these chromatographic parameters show that the methodology has been suitabialy optimised, with total run time under 8 min.

#### 3.3.2. Microsomal Incubations Procedure

Microsomal protein (0.5 mg/mL) was incubated at 37 °C, with a serial range of dexamethasone (10, 20, 30, 40, and 50 µM), magnesium chloride (3.0 mM), NADPH (1.0 mM), Glucose-6-Phosphate (5 mM), (Glucose-6-Phosphate Dehydrogenase (1.7 units/mL), Ethylenediaminetetraacetic acid (1.0 mM EDTA), and 0.067 M potassium phosphate buffer (pH 7.4) in a final volume of 500 µL. A serial range of aspirin (0, 50, 100, and 200 µM, dissolved in mobile phase) was added to the incubation mixture in triplicate. Incubations were for 40 min and were initiated by the addition of NADP^+^ (Nicotinamide Adenine Dinucleotide Phosphate) to the mixture after the pre-incubation of all of the components for 5 min in a water bath (T = 37 °C).

Ice-cold-grade acetonitrile containing 15 µM 4-hydroxyoctanophenone (as an internal standard) was added to terminate the reaction. Dexamethasone and metabolite were extracted with ethyl acetate (3 mL) and then with diethyl ether (3 mL). The polar extracts were evaporated to dryness, and the residues were dissolved in the mobile phase (70% acetonitrile and 30% water, *v/v*) and made up to 1000 µL in volume. An amount of 10 µL of each sample was injected into the HPLC instrument for analysis.

### 3.4. Preparation of Standard Substrate and Metabolite Solutions

For the cytochrome P3A2 enzyme assay, 4-hydroxyoctanophenone was used as an internal standard. An amount of 0.0010 g of the powder was dissolved in acetonitrile in a 10 mL volumetric flask. The final stock (15 µM) was prepared by adding 165 µL of the stock in a volumetric flask containing 49 mL and 835 µL of mobile phase (70% methanol + 30% water, *v/v*).

Aspirin (0.0018 g) (C = 1000 µM) was weighed accurately and dissolved in a 10 mL volumetric flask in methanol. Serial dilutions of aspirin (200, 100, and 50 µM) were performed. An amount of 0.0039 g of dexamethasone (C = 1000 µM) was weighed and added to a 10 mL volumetric flask containing methanol. Different concentrations of dexamethasone (50, 40, 30, 20, and 10 µM) from the stock solution were prepared in the mobile phase (70% methanol + 30% water, *v/v*). The metabolite (6β-hydroxydexamethasone) stock solution of 2 µM was prepared in the mobile phase (70% methanol + 30% water, *v/v*), and serial dilutions (0.2, 0.4, 0.6, 0.8, and 1 µM) were prepared from the stock.

### 3.5. Optimisation of Incubation Time In Vitro

The dexamethasone (substrate) and protein conentrations were fixed in the incubation system, and samples were incubated for 10, 20, 30, 40,50, 60, 70, and 100 min. The samples were prepared and incubated as described in Section 3.3. The concentrations of the produced metabolite 6β-hydroxydexamethasone were calculated from the standard calibration curve. The optimal incubation time was determined by the linear relationship between the time and metabolite concentration.

### 3.6. Data Analysis

Data were analysed using Microsoft Excel 2010 software for validation and the kinetic parameters. All results are shown as mean ± S.D. The least-square regression analysis was performed to calculate the concentration of the metabolite produced by the CYP reaction. Secondary Lineweaver–Burk plots and Michaelis–Menten plots were plotted to find kinetic parameters such as K_i_, V_max_, K_m_, Cl_int,_ and α^,^. Inhibition was assumed to be competitive based on the obtained data and based on a visual inspection of the Lineweaver–Burk plot. The inhibitor concentration that would cause a 50% reduction in enzyme activity (IC_50_) was calculated by plotting the percentage of the remaining control enzyme activity versus the inhibitor concentration. *K*_m_ values were used to calculate the percentage of inhibition.

## 4. Conclusions

In conclusion, a HPLC method was developed, and analytical parameters including linearity, precision, % recovery, linear regression, LOD, and LOQ were derived for dexamethasone and 6β-hydroxydexamethasone. All of the analytical parameters were validated in accordance with the ICH guidelines. In vitro incubation assays using rat liver microsomes were adopted to determine the effects of aspirin on dexamethasone metabolism (CYP3A2 activity), as aspirin and dexamethasone have been used together for COVID-19 treatment. Our findings revealed that aspirin acts as a competitive inhibitor and that it has a weak inhibitory effect on dexamethasone metabolism in rat liver microsomes. The outcomes of the study further suggest the safe use of aspirin and dexamethasone in clinical practice. Nevertheless, further in vivo inhibition studies are required to consider this interaction and its implications more completely for patient care.

## Figures and Tables

**Table 1:** Linearity data from the proposed analytical method.

Standards	Dexamethasone	6β-Hydroxydexamethasone
Regression equation	y = 0.2505x + 0.0945	y = 1.6775x + 0.0385
r^2^	0.999	0.998
Linear range	25–200 µM	0.2–1 µM

**Table 2:** LOD and LOQ for dexamethasone and 6β-hydroxydexamethasone.

Standards	Dexamethasone	6β-Hydroxydexamethasone
LOD	5.60 µM	0.06 µM
LOQ	16.98 µM	0.19 µM

**Table 3:** Intraday precision and recovery for dexamethasone (*n* = 3).

Dexamethasone Standards	Mean (µM)	Recovery ^a^ (%)	RSD (%)
Low concentration (40 µM)	39.28 ± 0.90	98.21	2.30
Medium concentration (110 µM)	99.06 ± 3.06	90.05	3.09
High concentration (185 µM)	151.11 ± 0.76	81.68	0.50

Note: Recovery is the ratio of the concentration of analyte recovered to the theoretical concentration. ^a^ % recovery = (concentration of dexamethasone at 5 h/standard concentration of dexamethasone) × 100.

**Table 4:** Interday precision and recovery for dexamethasone (*n* = 3).

Dexamethasone Standards	Mean (µM)	Recovery ^a^ (%)	RSD (%)
Low concentration (40 µM)	45.01 ± 2.09	112.52	4.65
Medium concentration (110 µM)	110.01 ± 2.17	100.01	1.98
High concentration (185 µM)	178.92 ± 3.13	96.72	1.75

Note: ^a^ % recovery = (concentration of dexamethasone at 5 h/standard concentration of dexamethasone) × 100.

**Table 5:** Intraday precision and recovery for 6β-hydroxydexamethasone (*n* = 3).

6β-Hydroxydexamethasone Standards	Mean (µM)	Recovery ^a^ (%)	RSD (%)
Low concentration (0.3 µM)	0.33 ± 0.01	108.60	2.21
Medium concentration (0.5 µM)	0.50 ± 0.02	100.01	3.64
High concentration (0.85 µM)	1.02 ± 0.09	119.38	8.82

Note: ^a^ % recovery = (concentration of 6β-hydroxydexamethasone at 5 h/standard concentration of 6β-hydroxydexamethasone) × 100.

**Table 6:** Interday precision and recovery for 6β-hydroxydexamethasone (*n* = 3).

6β-Hydroxydexamethasone Standards	Mean (µM)	Recovery ^a^ (%)	RSD (%)
Low concentration (0.3 µM)	0.32 ± 0.02	107.61	4.67
Medium concentration (0.5 µM)	0.50 ± 0.03	99.28	5.90
High concentration (0.85 µM)	0.79 ± 0.03	93.13	4.13

Note: ^a^ % recovery = (concentration of 6β-hydroxydexamethasone at 5 h/standard concentration of 6β-hydroxydexamethasone) × 100.

**Table 7:** Dexamethasone solutions stability at ambient temperature.

Analytical Parameters	Actual Concentration (µM)			
	Intraday	40	110	185
**Calculated Concentration** **(** **µ** **M)**	0 h	35.40	88.64	176.98
	5 h	32.20	92.43	202.56
	10 h	32.32	92.87	203.56
**% Recovery**	0 h	88.51	80.58	95.66
	5 h	80.50	84.03	109.49
	10 h	80.80	84.43	110.03
**% Accuracy** **^a^**	0 h	111.49	119.42	104.34
	5 h	119.51	115.97	90.51
	10 h	119.20	115.57	89.97
**Calculated Concentration** **(** **µ** **M)**	**Interday**	**40**	**110**	**185**
	Interday 1	32.02	94.39	181.79
	Interday 2	34.99	100.43	193.84
	Interday 3	34.12	90.60	187.22
**% Recovery**	Interday 1	80.04	85.81	98.26
	Interday 2	87.31	91.30	104.78
	Interday 3	85.29	82.36	101.20
**% Accuracy ^a^**	Interday 1	119.96	114.19	101.74
	Interday 2	112.70	108.70	95.22
	Interday 3	114.71	117.64	98.80

Note: ^a^ % Accuracy = 100 − (calculated concentration − actual concentration/actual concentration) × 100.

**Table 8:** 6β-hydroxydexamethasone solutions stability at ambient temperature.

Analytical Parameters	Actual Concentration (µM)			
	Intraday	0.3	0.5	0.85
**Calculated Concentration** **(** **µ** **M)**	0 h	0.30	0.47	0.84
	5 h	0.29	0.44	0.77
	10 h	0.27	0.44	0.79
**% Recovery**	0 h	98.18	94.82	99.34
	5 h	97.22	87.58	90.45
	10 h	88.89	87.75	92.98
**% Accuracy ^a^**	0 h	101.82	105.18	100.66
	5 h	102.79	112.42	109.55
	10 h	111.11	112.25	107.02
	**Interday**	**0.3**	**0.5**	**0.85**
**Calculated Concentration** **(** **µ** **M)**	Interday 1	0.27	0.46	0.72
	Interday 2	0.32	0.47	0.69
	Interday 3	0.32	0.46	0.68
**% Recovery**	Interday 1	89.49	92.64	84.06
	Interday 2	105.46	94.15	81.57
	Interday 3	106.90	92.10	80.51
**% Accuracy ^a^**	Interday 1	110.51	107.36	115.94
	Interday 2	94.54	105.85	118.43
	Interday 3	93.10	107.90	119.49

Note: ^a^ % Accuracy = 100 − (calculated concentration − actual concentration/actual concentration) × 100.

**Table 9:** Evaluation of robustness parameters: (A) Change in temperature; (B) change in wavelength; (C) Change in flow rate.

Analytes of Interest	Average t_R_	Average Peak Area	Resolution
	Normal Conditions (0.6 mL/min, 243 nm and 25 °C)		
**Aspirin**	2.04	16,162.67	All compounds were well separated, and a good resolution was achieved.
**6** **β-Hydroxydexamethasone**	2.64	127,567.67	
**Dexamethasone**	3.08	58,676.00	
**4-Hydroxyoctanophenone**	6.67	31,991.33	
	**A: Temperature (30 °C)**		
**Aspirin**	2.04	12,351.00	All compounds were separated well, with a faster elution pattern as the temperature increased.
**6** **β-Hydroxydexamethasone**	2.64	120,462.67	
**Dexamethasone**	3.07	55,890.67	
**4-Hydroxyoctanophenone**	6.51	29,137.67	
	**B: Wavelength (248 nm)**		
**Aspirin**	2.08	10,039.33	All four compounds were separated, but there was a decrease in intensity of metabolite peak.
**6** **β-Hydroxydexamethasone**	2.67	75,995.00	
**Dexamethasone**	3.08	49,674.00	
**4-Hydroxyoctanophenone**	6.61	53,310.00	
	**C: Flow rate (0.8 mL/min)**		
**Aspirin**	1.42	6458.00	Peaks were separated with a 0.8 mL/min flow rate. All compounds showed a faster and narrow elution pattern.
**6** **β-Hydroxydexamethasone**	1.99	97,253.00	
**Dexamethasone**	2.33	38,095.00	
**4-Hydroxyoctanophenone**	5.02	22,253.33	

**Table 10:** Pharmacokinetic parameters of inhibitory effects of aspirin on enzyme metabolism. The mean result is taken ± SD (*n* = 3). Note: *p* < 0.001.

Aspirin Concentration	(Inhibition Parameters)				
	K_m_ (µM)	V_max_ (µM^−1^∙min^−1^)	Cl_int_ (µM^−2^∙min^−1^)	á	%Inhibition
**0 µM Aspirin**	21.23 ± 0.51	0.0127 ± 1.53 × 10^−4^	0.0006 ± 1.10 × 10^−5^	-	-
**50 µM Aspirin**	23.83 ± 0.31	0.0123 ± 1.15 × 10^−4^	0.0005 ± 4.00 × 10^−6^	1.03 ± 0.01	12.44 ± 1.20
**100 µM Aspirin**	26.13 ± 0.70	0.0127 ± 7.94 × 10^−4^	0.0005 ± 5.00 × 10^−5^	1.02 ± 0.06	23.29 ± 3.30
**200 µM Aspirin**	32.57 ± 0.35	0.0123 ± 1.73 × 10^−4^	0.0004 ± 1.00 × 10^−5^	1.04 ± 0.01	53.64 ± 1.76

**Table 11:** Column efficiency, plate height, asymmetry, and resolution of compounds of interest.

	Aspirin		6β-Hydroxydexamethasone		Dexamethasone		Internal Standard	
**Retention Time (t_R_)**	2.09		2.65		3.08		6.66	
**Efficiency (N)**	1078		970		3363		6137	
**Plate Height (H)**	1.39 × 10^−2^		1.55 × 10^−2^		4.46 × 10^−3^		2.44 × 10^−3^	
**Resolution (R_s_)**		1.58		1.33		11.01		
**Asymmetry Factor** (**AsF)**	0.97		1.03		1.07		1.07	

## Data Availability

The data presented in this study are available in this article.

## Supplementary Materials

The following supporting information can be downloaded, Figure S1: Typical HPLC chromatogram of CYP3A2 assay components obtained with 0.8 mL/min flow rate. The peaks marked are: (1) aspirin, (2) 6β-hydroxydexamethasone, (3) dexamethasone, (4) internal standard, respectively.

## Sample Availability

Samples of the compounds are available from the authors.

## References

[1] Bibi Z. (2008). Role of cytochrome P450 in drug interactions. Nutr. Metab..

[2] Lynch T., Price A. (2007). The Effect of Cytochrome P450 Metabolism on Drug Response, Interactions, and Adverse Effects. Am. Fam. Physician.

[3] Ko J.W., Desta Z., Soukhova N.V., Tracy T., Flockhart D.A. (2000). In Vitro inhibition of the cytochrome P450 (CYP450) system by the antiplatelet drug ticlopidine: Potent effect on CYP2C19 and CYP2D6. Br. J. Clin. Pharmacol..

[4] Nduka S.O., Okonta M.J., Ajaghaku D.L., Ukwe C.V. (2017). In Vitro and in vivo cytochrome P450 3A enzyme inhibition by Aframomum melegueta and Denniettia tripetala extracts. Asian Pac. J. Trop..

[5] McDonnell A.M., Dang C.H. (2013). Basic Review of the Cytochrome P450 System. J. Adv. Pract. Oncol..

[6] Sprouse A.A., van Breemen R.B. (2016). Pharmacokinetic interactions between drugs and botanical dietary supplements. Drug Metab. Dispos..

[7] Zurbonsen K., Bressolle F., Solassol I., Aragon P.J., Culine S., Pinguet F. (2004). Simultaneous determination of dexamethasone and 6β-hydroxydexamethasone in urine using solid-phase extraction and liquid chromatography: Applications to in vivo measurement of cytochrome P450 3A4 activity. J. Chromatogr. B.

[8] Ahmed M.H., Hassan A. (2020). Dexamethasone for the Treatment of Coronavirus Disease (COVID-19): A Review. SN Compr. Clin. Med..

[9] Al Rihani S.B., Deodhar M., Dow P., Turgeon J., Michaud V. (2020). Is Dexamethasone a Substrate, an Inducer, or a Substrate-Inducer of CYP3As?. Arch. Pharm. Res..

[10] Park B.K., Back D.J. (1996). Dexamethasone metabolism by human liver in vitro. Metabolite identification and inhibition of 6-hydroxylation. J. Pharmacol. Exp. Ther..

[11] Tomlinson E.S., Maggs J.L., Park B.K., Back D.J. (1997). Dexamethasone Metabolism in Species Differences. J. Steroid Biochem. Mol..

[12] Wang S., Dong Y., Su K., Zhang J., Wang L., Han A., Wen C., Wang X., He Y. (2017). Effect of codeine on CYP450 isoform activity of rats. Pharm. Biol..

[13] Hector A.C., Roberto H., Eugenia F.M., Muniz F.J., Aires B. (2020). Safety and efficacy of the combined use of Ivermectin, Dexamethasone, Enoxaparin and Aspirin, against Covid 19. medRxiv.

[14] Vukoja D., Juric A., Erkapic Z., Pejic T., Zovko Z., Juricic J., Pejic J., Corluka M. (2021). Beneficial Treatment Outcomes of Severe COVID-19 Patients Treated Entirely in Primary Care Settings With Dexamethasone Including Regimen—Case Series Report. Front. Pharmacol..

[15] Pan Z., Camara B., Gardner H.W., Backhaus R.A. (1998). Aspirin Inhibition and Acetylation of the Plant Cytochrome P450, Allene Oxide Synthase, Resembles that of Animal Prostaglandin Endoperoxide H Synthase. Am. Soc. Biochem. Mol..

[16] Ornelas A., Zacharias-Millward N., Menter D.G., Davis J.S., Lichtenberger L., Hawke D., Hawk E., Vilar E., Bhattacharya P., Millward S. (2017). Beyond COX-1: The effects of aspirin on platelet biology and potential mechanisms of chemoprevention. Cancer Metastasis Rev..

[17] Vane J.R., Botting R.M. (2003). The mechanism of action of aspirin. Thromb. Res..

[18] (2005). International Conference on Harmonisation of Technical Requirements for Registration of Pharmaceuticals for Human Use. ICH Harmonised Tripartite Guideline, Validation of Analytical Procedures: Text and Methodology Q2(R1).

[19] Heda A.A., Kathiriya J.M., Gadade D.D., Puranik P.K. (2011). Development-and-validation-of-rphplc-method-for-simultaneous-determination-of-granisetron-and-dexamethasone. Indian J. Pharm. Sci..

[20] Jafari-Fesharaki M., Scheinman M.M. (1998). Adverse Effects of Amiodarone. Pacing Clin. Electrophysiol..

[21] Strelow J., Dewe W., Iversen P.W., Brooks H.B., Radding J.A., McGee J., Weidn J. (2012). Mechanism of Action Assays for Enzymes. AGM.

[22] Liu L.I., Jiang Z., Liu J., Huang X., Wang T., Liu J., Zhang Y., Zhou Z., Guo J., Yang L. (2010). Sex differences in subacute toxicity and hepatic microsomal metabolism of triptolide in rats. Toxicology.

[23] Zhang Z.J., Xia Z.Y., Wang J.M., Song X.T., Wei J.F., Kang W.Y. (2016). Effects of flavonoids in Lysimachia clethroides duby on the activities of cytochrome P450 CYP2E1 and CYP3A4 in rat liver microsomes. Molecules.

