# Supplementary material for: Development and Validation of a Novel HPLC Method to Analyse Metabolic Reaction Products Catalysed by the CYP3A2 Isoform: In Vitro Inhibition of CYP3A2 Enzyme Activity by Aspirin (Drugs Often Used Together in COVID-19 Treatment)
Source: Molecules. 2022 Jan 29;27(3):927. doi: 10.3390/molecules27030927 (PMC8838585; doi:10.3390/molecules27030927)
Supplement: Supplementary file 1 [file molecules-27-00927-s001.zip › molecules-1477175-supplementary.pdf]

Supplementary information

Robustness parameter (C) Change in flow rate (0.8 mL/min)

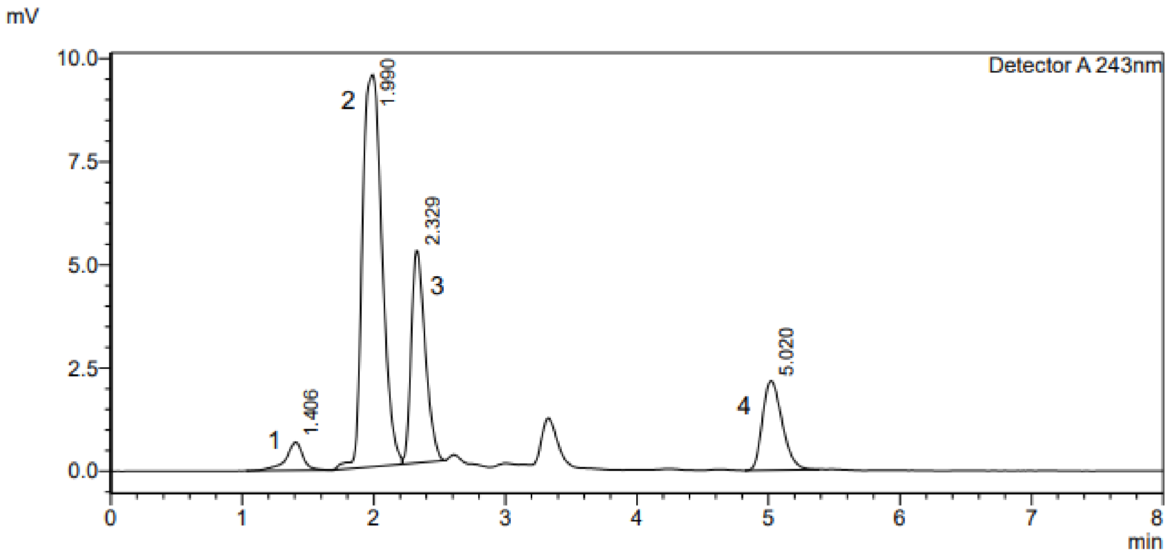

Figure S1. Typical HPLC chromatogram of CYP3A2 assay components obtained with 0.8 mL/min flow rate. The peaks marked are: (1) aspirin, (2) 6β-hydroxydexamethasone, (3) dexamethasone, (4) internal standard, respectively.
